# Genomes of three root-associated bacteria isolated from *Sorghum bicolor* L. Moench under arid-land conditions

**DOI:** 10.1128/mra.00401-25

**Published:** 2025-09-12

**Authors:** Ciara Garcia, Duke Pauli, Dave Baltrus, A. Elizabeth Arnold

**Affiliations:** 1 School of Plant Sciences, University of Arizona 124485, Tucson, Arizona, USA; 2 Center for Agroecosystem Research in the Desert (ARID), University of Arizona 8041, Tucson, Arizona, USA; 3 BIO5 Institute, University of Arizona 124486, Tucson, Arizona, USA; 4 Department of Ecology and Evolutionary Biology, University of Arizona 8041, Tucson, Arizona, USA; University of Strathclyde, Glasgow, United Kingdom

**Keywords:** agriculture, sorghum, rhizosphere, epiphytic, plant-associated bacteria

## Abstract

Diverse Proteobacteria and Actinobacteria affiliate with plants, including strains associated with plant growth and resilience. Here, we share annotated genome sequences for three root-associated bacteria isolated from *Sorghum bicolor* L. Moench in Arizona: *Sphingomonas* sp. and *Roseateles* sp. (Proteobacteria), and *Promicromonospora* sp. (Actinobacteria).

## ANNOUNCEMENT

Members of the Proteobacteria and Actinobacteria comprise a large proportion of most plant microbiomes (1, 2). Sorghum, a cereal grain that can be grown in arid regions, is an emerging model crop for stress resilience. Root-associated microbial communities differ among sorghum genotypes, aligning with cultivar-specific productivity (3). Here, we present three genome sequences from root-associated bacteria isolated from sorghum in arid-land agriculture conditions. This work contributes to insights linking crop performance and genomic features of the microbiome.

Isolates were originally obtained in 2021–2022 from roots of high-performing (*Sphingomonas* sp. and *Promicromonospora* sp.) and low-performing sorghum (*Roseateles* sp.) collected at the Maricopa Agricultural Center in Arizona, USA (33°04′37″N, 111°58′26″W) (3). Isolates from this culture collection were streaked from glycerol stocks stored at −80°C (3) onto Luria broth Agar and grown at 27°C in aerobic conditions for 48–72 h. Isolated colonies were grown in 4 mL of Luria Broth for 40 h at 150 rpm to reach ca. 10^8^ CFU. Cultures were harvested entirely for DNA extraction with the Wizard Genomic DNA Isolation Kit (Promega, Wisconsin, USA; manufacturer’s protocol). Optional incubation with lysozyme ensured efficient lysis for *Promicromonospora*. DNA was eluted in 40 µL of elution buffer (10 mM Tris, pH 8.5, 0.1 mM ethylenediaminetetraacetic acid [EDTA]). DNA quantity and qualitywere assessed with a NanoDrop 2000 (Thermo Scientific). Genomic DNA was diluted to 50 ng/µL in nuclease-free water and sequenced by Plasmidsaurus (Eugene, OR, USA) via hybrid Oxford Nanopore Technology (ONT) long-read+Illumina short-read whole-genome sequencing. An amplification-free long-read sequencing library was prepared with the ONT Rapid Barcoding Kit 96 v14 without fragmentation or size selection and sequenced on the PromethION P24 using an R10.4.1 flow cell and ont-dorado-for-promethion v0.5.0 with super-accurate as the base-calling mode (https://github.com/nanoporetech/dorado). The 5% worst fastq reads were filtered with Filtlong v0.2.1 (see 4). Reads were downsampled to 250 Mb and a draft genome was assembled with Flye v2.9.1 with parameters for high-quality ONT reads, then polished via Medaka v1.8.0. An additional library was prepared from the same gDNAs with an ExpressPlex 96 Library Prep Kit (SeqWell) and sequenced on an Illumina NextSeq2000 with paired-end 2 × 150 bp chemistry. Unfiltered .fastq reads were used to polish the ONT .fna assembly via Polypolish v0.6.0 (5), yielding a .fasta file reflecting hybrid assembly. One closed circular contig was produced by each assembly. Genome completeness and contaminationwere assessed with CheckM v1.2.2. Unless otherwise noted, default parameters were used for all software.

The most closely related type strains identified via the Type Strain Genome Server (TYGS) v400 (6) were used to calculate average nucleotide identity (ANI) via EZ Bio Cloud’s orthologous ANI tool (https://www.ezbiocloud.net/tools/ani). We detected 84.7% ANI between *Sphingomonas* sp. (MS122) and *Sphingomonas koreensis* NBRC16723, 86.3% ANI between *Roseateles* sp. (MS654) and *Roseateles chitinovorans* HWN-4, and 86.1% ANI between *Promicromonospora* sp. (MS192) and *Promicromonospora thailandica* DSM26652. Assembled genomes were uploaded to NCBI (Table 1) and annotated with PGAP v6.10 (7). Annotation with antiSMASH v8.0 (8) indicated that *Promicromonospora* sp. and *Roseateles* sp. have genes predicted to encode for acyl amino acids and siderophores. *Promicromonospora* sp. also has genes predicted to encode for ectoine, an extremolyte that confers resistance to salt and temperature stress (9).

**TABLE 1:** Assembly and genomic characteristics for three *Sorghum* root-associated bacteria^*a*^

Species	*Sphingomonas* sp.	*Roseateles* sp.	*Promicromonospora* sp.
Strain ID	MS122	MS654	MS192
GenBank accession	CP186695	CP186694	CP186693
Genome size (Mbp)	4.11	5.79	5.99
ONT raw reads	27,211	26,008	41,736
Illumina raw reads	322,167	1,127,045	643,642
N50 (kb)	14.28	19.95	20.46
Coverage from hybrid assembly	39×	36×	52×
CDS	3,869	4,891	5,271
tRNAs	46	63	51
GC Content	67.9	68.2	72.9
Completeness (%)	98.92	99.84	100.00
Contamination (%)	1.42	0.93	0.00

^*a*^ Taxonomy was inferred from TYGS. Completeness and contamination reflect values obtained from CheckM.

## Data Availability

Assembled genomes are listed in Table 1. Raw reads have been deposited in the Sequence Read Archive under the BioProject accession PRJNA1252778 under accessions SRX28466841, SRX28466843, and SRX28466845 for ONT reads, and under accessions SRX28466842, SRX28466844, and SRX28466846 for Illumina reads.
